# Slipped capital femoral epiphysis after treatment of femoral neck fracture

**DOI:** 10.12669/pjms.36.ICON-Suppl.1725

**Published:** 2020-01

**Authors:** Muhammad Amin Chinoy, Sateesh Pal, Mansoor Ali Khan

**Affiliations:** 1 Muhammad Amin Chinoy, FRCS. Department of Orthopedics, The Indus Hospital, Karachi, Pakistan; 2 Sateesh Pal, FCPS. Department of Orthopedics, The Indus Hospital, Karachi, Pakistan; 3 Mansoor Ali Khan, FCPS. Department of Orthopedics, The Indus Hospital, Karachi, Pakistan

**Keywords:** Avascular necrosis, Coxa vara, Femur neck fracture, Slipped capital femoral epiphysis

## Abstract

Slipped capital femoral epiphysis (SCFE) in children after treatment of femoral neck fracture is a very rare condition. This complication should be recognized promptly and treated urgently. The risk of development of this complication can be minimized by anatomical reduction of the fracture and stable internal fixation of the fracture. Five years old male child sustained right sided femur neck fracture and was treated with closed reduction and Hip spica cast application. The fracture healed with a varus deformity. After 7 months, he developed slip of femoral epiphysis with a coxa vara deformity of proximal femur, which was treated with in situ fixation with Cannulated screws. His subsequent course remained uneventful up to five months. Slipped capital femoral epiphysis (SCFE) after treatment of femoral neck fracture in children is a rare complication that should be recognized and treated promptly. The onset of SCFE may show inadequate reduction or fixation of the fracture. Anatomic reduction and stable internal fixation for femoral neck fracture in children provides best outcomes. Postoperative care and delayed weight bearing are also equally important to avoid complications.

## INTRODUCTION

Slipped capital femoral epiphysis (SCFE) in children after treatment of femoral neck fracture is a very rare condition that poses important treatment challenges^1^ Femoral neck fractures in children are associated with high incidence of complications like avascular necrosis of femoral head, delayed union, mal-union and non-union, so they need to be treated early to reduce the risk of complications.^2^ The adequacy of fracture reduction and timing of treatment from the injury plays an important role in the outcome of femoral neck fractures in children.^3^

## CASE REPORT

**Fig.1:**
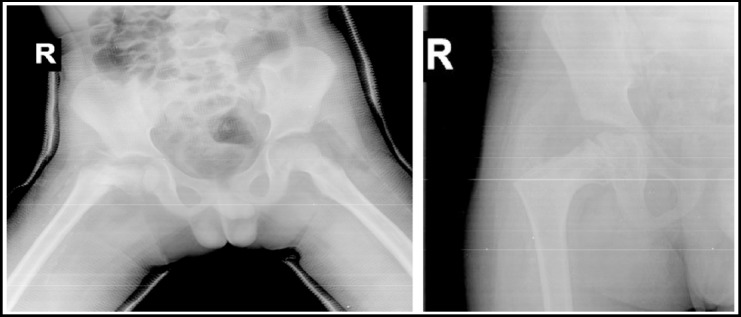
X-rays after spica application and 3 months post op.

**Fig.2:**
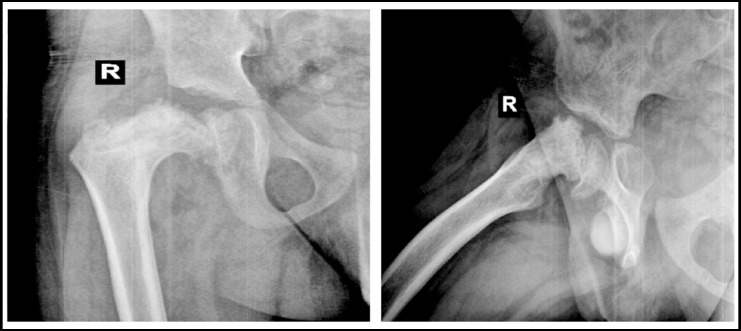
X-rays after 7 months of spica.

**Fig.3:**
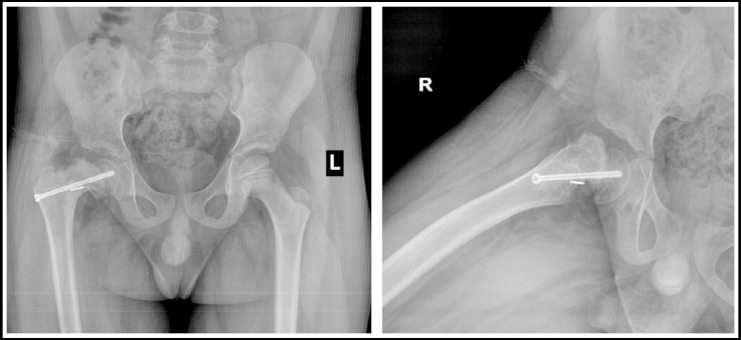
X-rays after fixation of slip with cannulated screw.

Five years old male child of average height and built with BMI of 21% presented with right sided femur neck fracture after sustaining a fall from height. The fracture was classified as per Delbet classification as type 3. He was treated with closed reduction under general anesthesia and Hip spica cast application (Fig.1). His serum vitamin D levels were 18ng/ml. The fracture healed with a varus deformity (neck shaft angle of 100 degrees). He was doing well up to seven months post surgery and was mobilized full weight bearing without support. After 7 months, he started feeling pain in his affected hip and started to limp. Examination revealed decreased hip movements with an external rotation deformity. The radiographs showed slip of femoral epiphysis with a coxa vara deformity of proximal femur. (Fig.2) His serum vitamin D levels were 18ng/ml and his endocrine work up reported to be normal. In situ fixation with Cannulated screws of the epiphyseal slip was performed. His subsequent course remained uneventful up to five months (Fig.3).

## DISCUSSION

Slipped capital femoral epiphysis (SCFE) after treatment of femoral neck fracture can be caused by inadequate treatment of the fracture. Orthopedic surgeons should be aware of this unusual complication so that they can promptly recognize this complication and treat it urgently.^1^

Our patient was a healthy normal child without any endocrine or genetic disorder. Furthermore, there was no evidence of SCFE on plain radiographs taken at the time of the initial injury. Thus, in our case fracture mal-union caused by inadequate fracture reduction was found to be the main contributing factor in development of epiphyseal slip

Li et al reported two cases, a 12 year old girl and a six year-old girl, who developed SCFE at 5 months and nine months, respectively, after screw fixation of femoral neck fracture. They identified clinical factors such as implant irritation, early return to weight bearing, delayed union or nonunion, coxa vara, and avascular necrosis to be possibly related to the development of the subsequent slip.^1^

Gopinathan et al reported a case of 10 years old boy who sustained bilateral femoral neck fractures and treated with closed reduction and internal fixation with percutaneous cannulated screws. Child developed non union and slipped capital femoral epiphysis on one side five months after the surgery. They identified precarious blood supply of the growing femoral head, nonunion at the fracture site and early weight bearing as possible causes for the development of SCFE. They managed non union and SCFE with open reduction and percutaneous Cannulated screw fixation and non-vascularized fibular graft.^4^

Manukaran reported a case of slipped capital femoral epiphysis in a nine years old boy 14 months after internal fixation of femur neck fracture by cancellous screws and identified tip of implant at epiphyseal plate to be responsible for the slip.^5^

Ok et al reported a case of a six year old boy who developed SCFE following mal-union of the femur sub-trochanteric fracture. They recognized the alteration of shear force on epiphyseal plate to be responsible for the development of SCFE.^6^ Ogden reported a case of SCFE 15 months later in a 11 year old boy who sustained femur neck fracture and treated in hip spica cast followed by sub-trochanteric valgus osteotomy due to delayed union and coxa vara deformity. They concluded that post traumatic angular deformities of femur near hip joint can predispose to the physeal slip and emphasized the significance of normal anatomic alignment of the proximal femur while treating proximal femur fractures.^7^

Jung et al reported a case of SCFE in a 11 years old boy who sustained a delbet type three femoral neck fracture, treated with open reduction and internal fixation with Cannulated screw.15 months after the surgery, epiphyseal slip was noted that was treated with fixation with Cannulated screws and sub-trochanteric valgus osteotomy.^8^ Lahoti et al reported two cases of Separation of the Proximal Femoral Epiphysis After De-rotation Varus Osteotomy of the Femur which is a very common procedure done for the management of developmental dysplasia of the hip. However this was not related to trauma, but specifically related to De-rotation Varus osteotomy, as an elective procedure, and may have a completely different etiology and pathogenesis, than the other cases described above.^9^

Finnegan et al in his review article tried to find etiological and pathogenic basis of this very rare occurrence but concluded that probably a multinational and multi-centric effort would be needed as the numbers reported so far were very small.^10^

Barring the two cases reported by Lahoti et al.^9^, only 8 cases have been reported in the English literature, interestingly, most of these cases have been reported from Asia with the exception of that reported by Ogden et al.^7^ Is there a geographical and genetic variation? It needs to be further investigated.

## CONCLUSION

Slipped capital femoral epiphysis (SCFE) after treatment of femoral neck fracture in children is a rare complication that should be recognized and treated promptly. The onset of SCFE may show inadequate reduction or fixation of the fracture. Anatomic reduction, and stable internal fixation for femoral neck fracture in children provides best outcomes. Postoperative care and delayed weight bearing are also equally important to avoid complications.

### Authors’ Contribution

**MAC:** Designed and edited the manuscript.

**SP:** Did data collection and manuscript writing.

**MAK:** Did review and final approval of manuscript.
